# On Transient Queue-Size Distribution in a Model of WSN Node with Threshold-Type Power-Saving Algorithm

**DOI:** 10.3390/s22239285

**Published:** 2022-11-29

**Authors:** Wojciech M. Kempa, Dariusz Kurzyk

**Affiliations:** 1 Department of Mathematics Applications and Methods for Artificial Intelligence, Faculty of Applied Mathematics, Silesian University of Technology, 23 Kaszubska Str., 44-100 Gliwice, Poland; 2 Institute of Theoretical and Applied Informatics, Polish Academy of Sciences, 5 Bałtycka Str., 44-100 Gliwice, Poland

**Keywords:** *N*-policy, power saving, queue size, transient state, wireless sensor network

## Abstract

This article proposes a queueing model of the operation of a wireless sensor network node, in which a threshold strategy for starting the node after a period of no transmission is used. In this model, transmission of packets is resumed when the number of packets in the accumulation buffer reaches a predefined level. In the literature, most of the results for models with limited access to the service station are obtained in equilibrium. In this paper, a formula for the Laplace transform of the transient queue-size distribution is obtained and written using the key input parameters of the system. The analytical apparatus uses the concept of the embedded Markov chain, the formula for total probability, renewal theory and some supporting algebraic results. Numerical examples are attached as well.

## 1. Introduction

As is commonly known, wireless sensor networks (WSNs) are widely used in monitoring of different real-life phenomena, e.g., air pollution, fire risk, road traffic, military operations and many others. Sensors (nodes), typically equipped with a non-rechargeable battery, are often located in inaccessible places in which their eventual can be difficult. Hence, the problem of power saving is one of the most important challenges in WSN development. Currently, many attempts at improving the energy efficiency and lifetime of WSNs are being undertaken (see, e.g., [1,2,3,4] for related problems). In [5], we can find the usage of game theory for maximizing WSN lifetime, while [6] presents a low-power cluster-based routing algorithm for WSN that is biologically inspired by bee colonies. An interesting adaptive parallel processing strategy for WSNs is proposed in [7]. In [8], we can find an example of a queue-based power optimization algorithm. Different aspects and models of congestion control in WSNs are proposed and discussed in [9,10].

In [11] (see also [12,13,14,15]), a threshold-type *N*-policy is proposed as a model of power-saving mechanisms. In this algorithm, after the busy period during which packets are being transmitted continuously, the transmission of data messages is resumed after the suspension period only after at least *N* packets have accumulated in the buffer, where *N* is predetermined. This type of energy-saving mechanism allows the threshold value *N* to be adjusted depending on the purpose of the network. For example, in the case of fire risk monitoring, the value of *N* should be low, while in the case when the network is used to inform about the number of free parking spaces in the vicinity of a large shopping center, the value of this parameter may be greater.

One of the key questions when any mechanism of limiting access to a service station is used in the queueing model is its impact on the state of the queue length.

We deal with a model of a single network node based on a finite-capacity queueing system with group Poisson arrivals and a threshold-type policy. In the literature, analytical results for operating characteristics of various types of queueing systems are devoted mainly to the steady state of the system. In practice, however, transient analysis of the system is often desired. Such a situation occur in the case of the investigation of the system just after its restart after repair or simultaneously with the application of a new control policy.

Moreover, statistical behavior of the system may be disturbed by phenomena such as interference or fade-out, which are common in wireless networks.

According to the above-mentioned observations, the main motivation of the paper is to find explicit formulae for queue-size distribution in the considered model in the transient (non-stationary) case. We are interested in representations written via key input system parameters, without defining any additional random walk. In consequence, it will be possible to use these formulae directly in numerical computations.

The main contribution of the paper is a compact-form representation for the LT (=Laplace transform) of the transient queue-size distribution. The theoretical apparatus combines the notion of the embedded Markov chain, some facts from renewal theory and linear algebra. Analytical formulae are illustrated in numerical examples.

The considered queueing model offers interesting application possibilities (especially in wireless computer and telecommunication networks, and in production systems). With knowledge about the probability distribution of the number of packets present in the system, one can try to select the threshold value *N* so that the probability of exceeding a certain predetermined value *r* of the number of packets is appropriately small, e.g., below 0.05. With the value of *N* determined in this way, it is now possible to assess the cost of the system operation for the so-designed energy-saving mechanism. Assuming that the unit cost of functioning during the buffer accumulation period is $c_{L}$, and the unit cost of functioning during the server busy period is equal to $c_{B}$, the unit cost of system operation is a linear combination with the form

$$\begin{array}{cc} & \alpha c_{L}+\left(1-\alpha \right)c_{B}, \end{array}$$

where α stands for the fraction of time during which the system is in the buffer-loading period.

One can find a review of the newest results relating to queueing models with different restriction types in the access to the service station in [16], where in all models, the supplementary variable technique is used. For example, in [17], an M/G/1-type model with Poisson arrivals and generally distributed processing times of the fault-tolerant system with server vacation policy and threshold-type waking up is developed by using the supplementary variable technique. In particular, the representation for the remaining time to repair is obtained. Significant theoretical work devoted to models with various types of server vacations can be found in [18], which is an extensive monograph focused on this subject. Stationary analysis of queueing models with the mechanism of *N*-policy can be found in [19,20]. In particular, in [19], joining strategies for retrial queueing models with multiple vacation policy, *N*-policy and server failures are studied. In [20], the problem of optimal balking strategies is analyzed in a retrial queue with threshold-type policy with multiple vacations. A queueing model with batch Markovian arrival process and generally distributed service times operating under *N*-policy is investigated in [21], where the solution of the cost optimization problem is also found. An inventory model with auto-correlated arrival process and *N*-policy is studied in [22], where the arriving customers are impatient. The representation for the joint probability distribution of the level of inventory and the number of customers in the system is obtained. In [23], performance and cost analysis are presented of a queueing model with *N*-policy and a repairable service station. In [24], customer balking behavior in queues with *N*-policy mechanisms and geometric balking are compared.

Transient analysis of the queue-size distribution in infinite-buffer models with *N*-policy and some additional service restrictions can be found in [25] (see also [26,27,28]). New results for finite-buffer models with *N*-policy were obtained in [29], where the Poisson and general independent arrival streams are considered. In [30] (see also [31]), non-stationary results were obtained for systems with single vacation and working vacation policies, respectively. Time-dependent analysis of a multiple vacation queueing model with a threshold restarting policy is proposed in [32] for studying the power-saving mechanisms of a wireless sensor network using the dynamic power management technique. The considered model consists of a busy state, wake-up state, shutdown state and inactive state. In [33], an approach based on the supplementary variables technique is used to obtain the queue-size distribution in the equilibrium of the GI/M/1-type model (in which, referring to Kendall’s notation, packets arrive according to a general independent input stream and service times are independent exponential random variables) with finite waiting room. A multi-server version of this system with *N*-policy is analyzed in [34]. Another restarting algorithm is considered in [35] for the discrete-time MAP/G/1 model (with Markov arrival process and generally distributed service times), where the idle server resumes its service only when the accumulated workload exceeds the predetermined level. In [36], an (N,T)-policy is considered in which the system restarts the operation as soon as *N* messages are present or the waiting time of the leading message reaches a fixed time T.

The remainder of this paper is organized according to the following scheme. In the next Section 2, the queueing model is described mathematically. In Section 3, we find the formulae for LTs of the queue-size distribution during the buffer loading period and state the representation for the period duration. In Section 4, we deal with the queue-size distribution in a single busy period. The key formulae, following from the results obtained in Section 2, Section 3 and Section 4, are presented in Section 5. Section 6 contains numerical examples and Section 6 briefly summarizes the paper.

## 2. Mathematical Description of the Model

In the paper, we deal with a queueing system with a single service station and a finite waiting room in which packets enter in batches (groups) according to a compound Poisson process with group rate λ (i.e., the mean number of groups of packets occurring per time unit equals λ) and are served individually with a CDF (=cumulative distribution function) F(·) of the service time. Each entering group (batch) consists of a random number of packets. The size of an individual batch is generally distributed with a sequence $(p_{k}),$ $\sum_{k=1}^{\infty }p_{k}=1,$ where $p_{j}$ denotes the probability that the arriving group consists of exactly *j* packets. The total number of packets simultaneously present in the system is bounded by a non-random value K≥2, i.e., the system contains a waiting room (buffer) with capacity K−1 packets that accumulates packets waiting for service start. One place is dedicated to a packet being processed. The service process is governed by FIFO (i.e., first in first out, in that packets are being served in the order of their appearance in the system), as well. It is assumed that the system starts with the functioning empty and processing is initialized simultaneous with the arrival of the group containing the *N*th packet (the *N*-policy), where 1≤N≤K. For example, if N=6 and the first three arriving groups contain 1, 4 and 7 packets, respectively, the service process starts simultaneous with the arrival of the third group.

When the server becomes idle, it is turned off; it is restarted if *N* packets accumulate in the waiting room (therefore, just after the arrival of the group containing the *N*th packet).

In the functioning of the system, we can identify buffer loading periods $L_{1},L_{2},\cdots ,$ followed by busy periods $B_{1},B_{2},$ ⋯, during which the queue becomes empty. It follows from the memoryless property of exponentially distributed interarrival times that the initial and completion epochs of successive busy periods are renewal moments. Therefore, $\left(L_{k}\right)$ and $(B_{k}),$ k=0,1,⋯ are sequences of independent random variables with the same CDFs in each sequence treated separately. Moreover, we identify $L_{k}$ or $B_{k},$ k≥1, with their duration.

Let us denote by X(t) the number of packets present in the system at time t, including the packet being served at this moment (if any). In the next sections, we obtain the closed-form formulae for the LTs of the queue-size distribution during single buffer loading and busy periods. Next, utilizing the independence of buffer loading periods and busy periods, and the fact that they follow one by one cyclically, we obtain the general formulae for the LT of the queue-size distribution at any time, applying the renewal theory approach.

## 3. Queue-Size Distribution during Buffer Loading Period

Consider first the case of the queue-size distribution during the first buffer loading period $L_{1}$ beginning at time t=0. Due to the Poisson-type arrival process, we obtain for t≥0

(1) $$\begin{array}{cc} & P\left\{X\left(t\right)=m,t\in L_{1}\right\}=I\left\{0\leq m\leq N-1\right\}\sum_{i=0}^{m}p_{m}^{i*}\frac{{\left(\lambda t\right)}^{i}}{i!}e^{-\lambda t}, \end{array}$$

where I{A} stands for the indicator of the random event A and $p_{i}^{j*}$ denotes the *i*th term of the *j*-fold convolution of the sequence $\left(p_{k}\right)$, itself defined as follows:

(2) $$\begin{array}{cc} & p_{0}^{0*}=1,\;p_{i}^{j*}=\sum_{r=0}^{i}p_{r}^{(j-1)*}p_{i-r},\;j\geq 1. \end{array}$$

Indeed, the sum on the right side of (1) expresses the probability that exactly *m* packets arrive up to the fixed time *t*.

Introducing the following notation:

(3) $$\begin{array}{cc} & {\tilde{q}}^{L}\left(s,m\right)\overset{def}{=}\int_{0}^{\infty }e^{-st}P\left\{\left(X(t)=m\right)\cap \left(t\in L_{1}\right)\right\}dt, \end{array}$$

where s>0, we obtain from (1)

(4) $$\begin{array}{cc} {\tilde{q}}^{L}\left(s,m\right) & =I\left\{0\leq m\leq N-1\right\}\sum_{i=0}^{m}p_{m}^{i*}\int_{0}^{\infty }e^{-(s+\lambda )t}\frac{{\left(\lambda t\right)}^{i}}{i!}dt\\ & =I\left\{0\leq m\leq N-1\right\}\sum_{i=0}^{m}p_{m}^{i*}\frac{\lambda^{i}}{{\left(\lambda +s\right)}^{i+1}}. \end{array}$$

Moreover, since each buffer loading period duration has an Erlang-*N* distribution with parameter λ (i.e., the distribution of *N* independent identically exponentially distributed random variables, here, *N* successive interarrival times), we obtain

(5) $$\begin{array}{cc} {\tilde{g}}^{L}\left(s\right) & =\int_{0}^{\infty }e^{-st}dG^{L}\left(t\right)\overset{def}{=}\int_{0}^{\infty }e^{-st}dP\left\{L_{k}<t\right\}\\ & =\sum_{i=1}^{N}\sum_{j=i-1}^{N-1}p_{j}^{(i-1)*}\sum_{r=N-j}^{\infty }p_{r}\int_{0}^{\infty }e^{-(s+\lambda )t}\frac{\lambda^{i}}{(i-1)!}t^{i-1}dt\\ & =\sum_{i=1}^{N}{\left(\frac{\lambda }{\lambda +s}\right)}^{i}\sum_{j=i-1}^{N-1}p_{j}^{(i-1)*}\sum_{r=N-j}^{\infty }p_{r}. \end{array}$$

## 4. Queue-Size Distribution during Busy Period

Let us assume that the processing of packets can be initialized with any possible number *n* of packets accumulated in the buffer, where 1≤n≤K. Let $Q_{n}^{B}\left(t,\cdot \right)$ be the time-dependent conditional queue-size distribution at time *t* during the first busy period $B_{1}$ of the system, namely,

(6) $$\begin{array}{cc} & Q_{n}^{B}\left(t,m\right)\overset{def}{=}P\left\{X\left(t\right)=m,\;t\in B_{1}|\;X\left(0\right)=n\right\}, \end{array}$$

where t>0 and 1≤m,n≤K. In (6), we assume, for simplicity, that $B_{1}$ begins at time t=0. Because the consecutive departure moments are renewal moments (see, e.g., [37]), by applying the formula of total probability with respect to the first service completion moment after t=0, we obtain the following system of equations:

(7) $$\begin{array}{cc} Q_{1}^{B}\left(t,m\right)= & \sum_{i=1}^{K-2}\sum_{j=0}^{i}\int_{0}^{t}p_{i}^{j*}\frac{{\left(\lambda x\right)}^{j}}{j!}e^{-\lambda x}Q_{i}^{B}\left(t-x,m\right)dF\left(x\right)\\ & +\sum_{i=K-1}^{\infty }\sum_{j=0}^{i}\int_{0}^{t}p_{i}^{j*}\frac{{\left(\lambda x\right)}^{j}}{j!}e^{-\lambda x}Q_{K-1}^{B}\left(t-x,m\right)dF\left(x\right)\\ & +\bar{F}\left(t\right)e^{-\lambda t}[I\left\{1\leq m\leq K-1\right\}\sum_{i=0}^{m-1}p_{m-1}^{i*}\frac{{\left(\lambda t\right)}^{i}}{i!}\\ & +I\left\{m=K\right\}\sum_{i=K-1}^{\infty }\sum_{j=0}^{i}p_{i}^{j*}\frac{{\left(\lambda t\right)}^{j}}{j!}], \end{array}$$

and, for 2≤n≤K,

(8) $$\begin{array}{cc} Q_{n}^{B}\left(t,m\right)= & \sum_{i=0}^{K-n-1}\sum_{j=0}^{i}\int_{0}^{t}p_{i}^{j*}\frac{{\left(\lambda x\right)}^{j}}{j!}e^{-\lambda x}Q_{n+i-1}^{B}\left(t-x,m\right)dF\left(x\right)\\ & +\sum_{i=K-n}^{\infty }\sum_{j=0}^{i}\int_{0}^{t}p_{i}^{j*}\frac{{\left(\lambda x\right)}^{j}}{j!}e^{-\lambda x}Q_{K-1}^{B}\left(t-x,m\right)dF\left(x\right)\\ & +\bar{F}\left(t\right)e^{-\lambda t}[I\left\{n\leq m\leq K-1\right\}\sum_{i=0}^{m-n}p_{m-n}^{i*}\frac{{\left(\lambda t\right)}^{i}}{i!}\\ & +I\left\{m=K\right\}\sum_{i=K-n}^{\infty }\sum_{j=0}^{i}p_{i}^{j*}\frac{{\left(\lambda t\right)}^{j}}{j!}], \end{array}$$

where $\bar{F}\left(t\right)\overset{def}{=}1-F\left(t\right)$.

Let us explain (7) and (8). The first summands on the right side of (7) and (8) illustrates the situation in which the waiting room does not become saturated before the first service completion epoch at time x<t, while the second one relates to the case where all places in the waiting room are occupied before x. The last summands on the right side of (7) and (8) illustrate the situation in which the service of the first packet finishes after time t. Let us note that the only difference between (7) and (8) is in the sum taken in (8) from i=0. Indeed, if the initial state of the waiting room equals at least two, then—if no new packets occur before the first service completion epoch - the first departure moment does not finish the busy period.

After introducing the following notations:

(9) $${\tilde{q}}_{n}^{B}(s,m)\overset{def}{=}\int_{0}^{\infty }e^{-st}Q_{n}^{B}(t,m)dt,$$

(10) $$\begin{array}{c} \;\;\;\;a_{n}\left(s\right)\overset{def}{=}\int_{0}^{\infty }e^{-(s+\lambda )t}\sum_{j=0}^{n}p_{n}^{j*}\frac{{\left(\lambda t\right)}^{j}}{j!}dF\left(t\right),\\ \;\;\;\;\;\;\;\;\;\;\;\theta_{n}\left(s,m\right)\overset{def}{=}\int_{0}^{\infty }e^{-(\lambda +s)t}\bar{F}\left(t\right)[I\left\{n\leq m\leq K-1\right\}\sum_{i=0}^{m-n}p_{m-n}^{i*}\frac{{\left(\lambda t\right)}^{i}}{i!} \end{array}$$

(11) $$\begin{array}{cc} & \;\;\;\;\;\;\;\;\;+I\left\{m=K\right\}\sum_{i=K-n}^{\infty }\sum_{j=0}^{i}p_{i}^{j*}\frac{{\left(\lambda t\right)}^{j}}{j!}]dt, \end{array}$$

where Re(s)>0, we can transform Equations (7)–(8) to a more compact form as follows:

(12) $${\tilde{q}}_{1}^{B}\left(s,m\right)=\sum_{i=1}^{K-2}a_{i}\left(s\right){\tilde{q}}_{i}^{B}\left(s,m\right)+{\tilde{q}}_{K-1}^{B}\left(s,m\right)\sum_{i=K-1}^{\infty }a_{i}\left(s\right)+\theta_{1}\left(s,m\right),$$

(13) $$\;\;{\tilde{q}}_{n}^{B}\left(s,m\right)=\sum_{i=0}^{K-n-1}a_{i}\left(s\right){\tilde{q}}_{n+i-1}^{B}\left(s,m\right)+{\tilde{q}}_{K-1}^{B}\left(s,m\right)\sum_{i=K-n}^{\infty }a_{i}\left(s\right)+\theta_{n}\left(s,m\right),$$

where 2≤n≤K.

Next, we are interested in rewriting Equations (12) and (13) in a specific form allowing for the application of an auxiliary algebraic result. Note that if we substitute ${\tilde{d}}_{n}^{B}\left(s,m\right)\overset{def}{=}{\tilde{q}}_{K-n}^{B}\left(s,m\right),$ where 0≤n≤K−1, we obtain from (12) and (13)

(14) $$\begin{array}{cc} & \;\;\;\;\;\;\;\;{\tilde{d}}_{K-1}^{B}\left(s,m\right)=\sum_{i=1}^{K-2}a_{i}\left(s\right){\tilde{d}}_{K-i}^{B}\left(s,m\right)+{\tilde{d}}_{1}^{B}\left(s,m\right)\sum_{i=K-1}^{\infty }a_{i}\left(s\right)+\theta_{1}\left(s,m\right), \end{array}$$

(15) $$\begin{array}{cc} & \sum_{k=-1}^{n}a_{k+1}\left(s\right){\tilde{d}}_{n-k}^{B}\left(s,m\right)-{\tilde{d}}_{n}^{B}\left(s,m\right)=\phi_{n}\left(s,m\right), \end{array}$$

where 0≤n≤K−2 and

(16) $$\begin{array}{cc} & \phi_{n}\left(s,m\right)\overset{def}{=}a_{n+1}\left(s\right){\tilde{d}}_{0}^{B}\left(s,m\right)-{\tilde{d}}_{1}^{B}\left(s,m\right)\sum_{i=n+1}^{\infty }a_{i}\left(s\right)-\theta_{K-n}\left(s,m\right). \end{array}$$

In [38], a system of type (15) with an infinite number of equations (n≥0) was considered. It was proven there that each of its solution can be represented in the form

(17) $$\begin{array}{cc} & {\tilde{d}}_{n}^{B}\left(s,m\right)=M\left(s,m\right)R_{n+1}\left(s\right)+\sum_{i=0}^{n}R_{n-i}\left(s\right)\phi_{i}\left(s,m\right), \end{array}$$

where the functional sequence $\left(R_{n}\left(s\right)\right)$ is defined in a recursive way by the sequence $\left(a_{n}\left(s\right)\right)$ as follows:

(18) $$\begin{array}{cc} & R_{0}\left(s\right)=0,\;R_{1}\left(s\right)=a_{0}^{-1}\left(s\right),\\ & R_{n+1}\left(s\right)=R_{1}\left(s\right)\left[R_{n}\left(s\right)-\sum_{i=0}^{n}a_{i+1}\left(s\right)R_{n-i}\left(s\right)\right], \end{array}$$

where n≥1.

Because the number of equations in (15) is finite, we can use (14) as a specific-type boundary condition to find M(s,m) and, in consequence, obtain a unique solution. Moreover, it is necessary to obtain the explicit formula for ${\tilde{d}}_{0}^{B}\left(s,m\right)$ and ${\tilde{d}}_{1}^{B}\left(s,m\right),$ occurring in (16). Below, we write M(s,m) and ${\tilde{d}}_{1}^{B}\left(s,m\right)$ as functions of ${\tilde{d}}_{0}^{B}\left(s,m\right)$; then, we eliminate ${\tilde{d}}_{0}^{B}\left(s,m\right)$ explicitly.

Substituting n=0 into (17) leads to

(19) $$\begin{array}{cc} & M\left(s,m\right)=a_{0}\left(s\right){\tilde{d}}_{0}^{B}\left(s,m\right). \end{array}$$

Next, substituting n=0 into (15), we obtain

(20) $$\begin{array}{cc} & {\tilde{d}}_{1}^{B}\left(s,m\right)=a_{0}^{-1}\left(s\right)\left[\phi_{0}\left(s,m\right)+{\tilde{d}}_{0}^{B}\left(s,m\right)\left(1-a_{1}\left(s\right)\right)\right] \end{array}$$

and hence

(21) $$\begin{array}{cc} & {\tilde{d}}_{1}^{B}\left(s,m\right)=\frac{{\tilde{d}}_{0}^{B}\left(s,m\right)-\theta_{K}\left(s,m\right)}{f(s)}, \end{array}$$

where $f\left(s\right)\overset{def}{=}\int_{0}^{\infty }e^{-st}dF\left(t\right).$

Introducing (19) and (20) in (17) and taking into consideration (16), we can express ${\tilde{d}}_{n}^{B}\left(s,m\right)$ for n≥1 as a function of ${\tilde{d}}_{0}^{B}\left(s,m\right).$ Indeed, we have

(22) $$\begin{array}{cc} & {\tilde{d}}_{n}^{B}\left(s,m\right)=\gamma_{n}\left(s\right){\tilde{d}}_{0}^{B}\left(s,m\right)+\eta_{n}\left(s,m\right),\;n\geq 0, \end{array}$$

where

(23) $$\begin{array}{cc} & \gamma_{n}\left(s\right)\overset{def}{=}a_{0}\left(s\right)R_{n+1}\left(s\right)+\sum_{i=0}^{n}R_{n-i}\left(s\right)\left[a_{i+1}\left(s\right)-\frac{1}{f(s)}\sum_{j=i+1}^{\infty }a_{j}\left(s\right)\right] \end{array}$$

and

(24) $$\begin{array}{cc} & \eta_{n}\left(s,m\right)\overset{def}{=}\sum_{i=0}^{n}R_{n-i}\left(s\right)\left[\frac{\theta_{K}\left(s,m\right)}{f(s)}\sum_{j=i+1}^{\infty }a_{j}\left(s\right)-\theta_{K-i}\left(s,m\right)\right]. \end{array}$$

Substituting now (22) into (14) and referring to (21), we eliminate ${\tilde{d}}_{0}^{B}\left(s,m\right)$ in the following way:

(25) $$\begin{array}{cc} & {\tilde{d}}_{0}^{B}\left(s,m\right)=\Pi_{1}\left(s,m\right)\Pi_{2}^{-1}\left(s\right), \end{array}$$

where

$$\begin{array}{cc} \Pi_{1}\left(s,m\right)\overset{def}{=} & \sum_{i=1}^{K-2}a_{i}\left(s\right)\eta_{K-i}\left(s,m\right)-\frac{\theta_{K}\left(s,m\right)}{f(s)} \end{array}$$

(26) $$\begin{array}{cc} & \;\;\;\;\;\;\times \sum_{i=K-1}^{\infty }a_{i}\left(s\right)+\theta_{1}\left(s,m\right)-\eta_{K-1}\left(s,m\right), \end{array}$$

(27) $$\begin{array}{cc} \;\;\;\;\;\;\;\Pi_{2}\left(s\right)\overset{def}{=} & \gamma_{K-1}\left(s\right)-\sum_{i=1}^{K-2}a_{i}\left(s\right)\gamma_{K-i}\left(s\right)-\frac{1}{f(s)}\sum_{i=K-1}^{\infty }a_{i}\left(s\right). \end{array}$$

Let us denote by $\chi_{n}^{B}\left(\cdot \right)$ the LST (Laplace–Stieltjes transform) of CDF of the busy period duration in the system that begins the operation with 1≤n≤K packets present in the waiting room. It is easy to see that for $\chi_{1}^{B}\left(s\right),\cdots ,\chi_{K}^{B}\left(s\right)$, the following system of equations can be written (compare (12) and (13)):

(28) $$\;\;{\tilde{\chi }}_{1}^{B}\left(s\right)=\sum_{i=1}^{K-2}a_{i}\left(s\right){\tilde{\chi }}_{i}^{B}\left(s\right)+{\tilde{\chi }}_{K-1}^{B}\left(s\right)\sum_{i=K-1}^{\infty }a_{i}\left(s\right)+f\left(\lambda +s\right),$$

(29) $${\tilde{\chi }}_{n}^{B}\left(s\right)=\sum_{i=0}^{K-n-1}a_{i}\left(s\right){\tilde{\chi }}_{n+i-1}^{B}\left(s\right)+{\tilde{\chi }}_{K-1}^{B}\left(s\right)\sum_{i=K-n}^{\infty }a_{i}\left(s\right),$$

where 2≤n≤K and $f\left(s\right)\overset{def}{=}\int_{0}^{\infty }e^{-st}dF\left(t\right).$ Note that if we put

(30) $$\begin{array}{cc} & {\tilde{\varkappa }}_{n}\left(s\right)\overset{def}{=}\left\{\begin{array}{cc} f(\lambda +s),\; & n=1,\\ 0,\; & n\geq 2, \end{array}\right. \end{array}$$

then we can obtain the solution of the systems (28) and (29) by using a step-by-step procedure explained in detail in (14)–(27), taking ${\tilde{\varkappa }}_{n}\left(s\right)$ instead of $\varkappa_{n}\left(s,m\right).$ Because in the original model with *N*-policy each busy period begins with exactly *N* packets present, the only representation we need is the formula for ${\tilde{\chi }}_{N}^{B}\left(s\right).$ Referring to (22), we have

(31) $$\begin{array}{cc} & {\tilde{\chi }}^{B}\left(s\right)\overset{def}{=}{\tilde{\chi }}_{N}^{B}\left(s\right)=\gamma_{K-N}\left(s\right){\tilde{\Pi }}_{1}\left(s\right)\Pi_{2}^{-1}\left(s\right)+{\tilde{\eta }}_{K-N}\left(s\right),\;n\geq 0, \end{array}$$

where

(32) $$\begin{array}{cc} & {\tilde{\eta }}_{n}\left(s\right)\overset{def}{=}\sum_{i=0}^{n}R_{n-i}\left(s\right)\left[a_{0}^{-1}\left(s\right){\left(1+a_{0}^{-1}\left(s\right)\right)}^{-1}{\tilde{\varkappa }}_{K}\left(s\right)-{\tilde{\varkappa }}_{K-i}\left(s\right)\right] \end{array}$$

and

(33) $$\begin{array}{cc} & {\tilde{\Pi }}_{1}\left(s\right)\overset{def}{=}\sum_{i=1}^{K-2}a_{i}\left(s\right){\tilde{\eta }}_{K-i}\left(s\right)+{\tilde{\eta }}_{1}\left(s\right)\sum_{i=K-1}^{\infty }a_{i}\left(s\right)+{\tilde{\varkappa }}_{1}\left(s\right)-{\tilde{\eta }}_{K-1}\left(s\right) \end{array}$$

where $\gamma_{n}\left(s\right)$ and $\Pi_{2}\left(s\right)$ are defined in (23) and (27), respectively.

## 5. Main Result

In this section, the key result, namely, the explicit representation for the LT of the queue-size distribution in the considered queueing system with the *N*-policy, is given and proven.

**Theorem** **1.** *The formula for the LT of the queue-size distribution of in the M/G/1/K-type system with threshold-type N-policy is as follows:*

(34) $$\begin{array}{cc} & \int_{0}^{\infty }e^{-st}P\left\{X\left(t\right)=m\right\}dt=\frac{{\tilde{q}}^{L}\left(s,m\right)+{\tilde{g}}^{L}\left(s\right){\tilde{q}}_{N}^{B}\left(s,m\right)}{1-{\tilde{g}}^{B}\left(s\right){\tilde{g}}^{L}\left(s\right)}, \end{array}$$

*where the representations for ${\tilde{q}}^{L}\left(s,m\right),$ ${\tilde{g}}^{L}\left(s\right),$ ${\tilde{q}}_{N}^{B}\left(s,m\right)={\tilde{d}}_{K-N}^{B}\left(s,m\right)$ and ${\tilde{g}}^{B}\left(s\right)$ are given in (4), (5), (22) and (31), respectively.*

**Proof.**  Applying the formula of total probability, we obtain

(35) $$\begin{array}{cc} & P\left\{X\left(t\right)=m\right\}=\sum_{i=1}^{\infty }\left(P\left\{X\left(t\right)=m,\;t\in L_{i}\right\}+P\left\{X\left(t\right)=m,\;t\in B_{i}\right\}\right). \end{array}$$

Because random variables $L_{k}$ and $B_{k},$ k≥1, are independent and have identical distributions (in each sequence separately), we obtain

(36) $$\begin{array}{cc} & P\left\{X\left(t\right)=m,\;t\in L_{i}\right\}=\int_{0}^{t}P\left\{X\left(t-y\right)=m,\;t-y\in L_{1}\right\}d{\left(G^{L}*G^{B}\right)}^{(i-1)*}\left(y\right), \end{array}$$

(37) $$\begin{array}{cc} & \;\;\;P\left\{X\left(t\right)=m,\;t\in B_{i}\right\}=\int_{0}^{t}P\left\{X\left(t-y\right)=m,\;t-y\in B_{1}\right\}d\left[{\left(G^{L}\right)}^{i*}*{\left(G^{B}\right)}^{(i-1)*}\right]\left(y\right). \end{array}$$

Indeed, $(G^{L}*G^{B}{)}^{(i-1)*}\left(\cdot \right)$ is a CDF of (i−1) complete cycles consisting of a buffer loading period followed by a busy period. Similarly, $\left[{\left(G^{L}\right)}^{i*}*{\left(G^{B}\right)}^{(i-1)*}\right]\left(\cdot \right)$ represents the duration of the time consisting of (i−1) complete cycles and one additional buffer loading period.

In consequence, taking LTs of (36) and (37), referring to (35), we obtain the conclusion (34) of the theorem. □

## 6. Numerical Examples

In this section, we illustrate analytical results via numerical examples motivated by the operation of a hypothetical wireless packet network with an energy-saving threshold-type mechanism. Assume that data packets arrive at the node operating under the *N*-policy. Taking K=8 and 250 B as a packet “size” unit, we consider the following two different probability distributions of batch sizes:

• $P_{1}:\;p_{1}=p_{2}=\frac{1}{2},\;p_{k}=0,\;k>2$;
• $P_{2}:\;p_{1}=p_{2}=p_{3}=\frac{1}{3},\;p_{k}=0,\;k>3$.

which gives the following arrival rate parameters: $\lambda_{1}=200$ (for $P_{1}$) and $\lambda_{2}=150$ (for $P_{2}$), respectively. Obviously, in practice, data packets have different sizes and do not occur in batches. Therefore, the distributions $P_{1}$ and $P_{2}$ allow for modeling different packets sizes in the arriving flow. According to $P_{1}$, packets of sizes 250 and 500 B enter with the same frequency. Similarly, in the case of $P_{2}$, data packets of sizes 250, 500 and 750 B arrive with the same frequency. Furthermore, let packets be transmitted with two different processing speeds 800 kb/s and 600 kb/s, according to exponential service time distribution; these correspond to the mean processing times 2.50 and 3.33 ms (parameters of exponentially distributed service times equal $\mu_{1}=400$ and $\mu_{2}=300,$ respectively).

**Figure 1:**
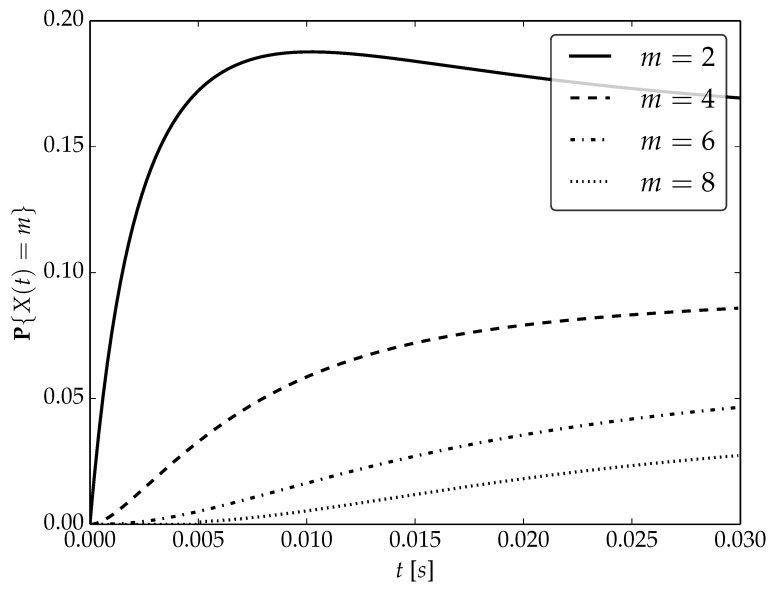
Transient distributions P{X(t)=m} for N=2, batch distribution $P_{1}$ and ρ=0.75.

**Figure 2:**
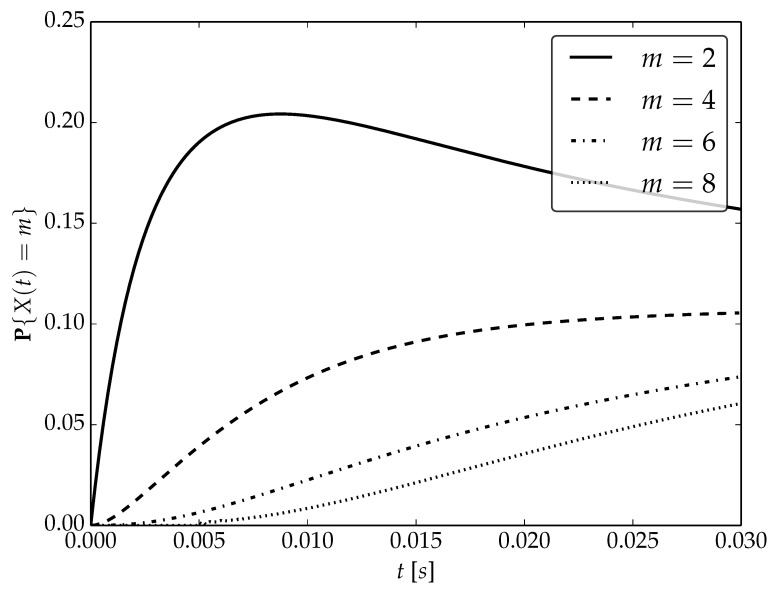
Transient distributions P{X(t)=m} for N=2, batch distribution $P_{1}$ and ρ=1.

**Figure 3:**
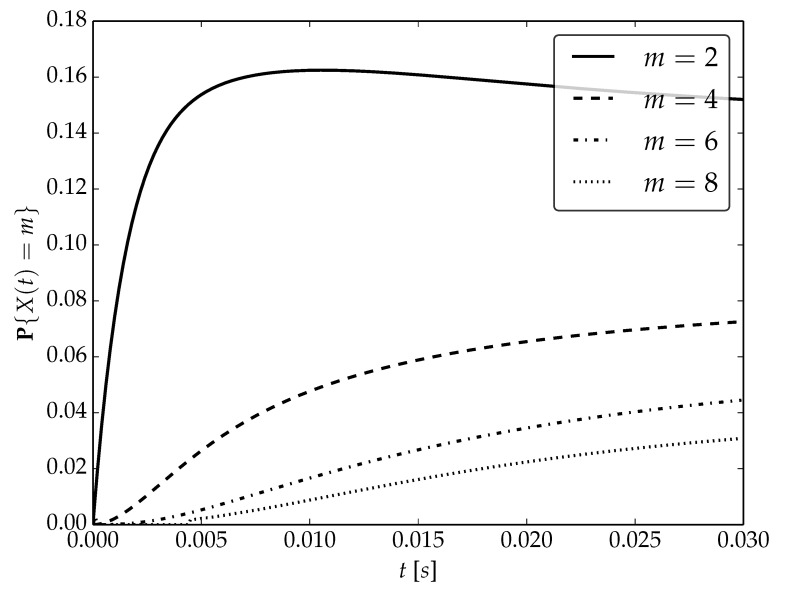
Transient distributions P{X(t)=m} for N=2, batch distribution $P_{2}$ and ρ=0.75.

Under the assumptions about arrival and serving rates, two different values of the utilization factor ρ of the considered system are possible, namely, 0.75 or 1. Transient probabilities P{X(t)=m} for ρ=0.75, ρ=1 and probability distributions $P_{1},P_{2}$ are visualized in Figure 1, Figure 2 and Figure 3 for N=2,4 and 6, respectively.

The obtained results show a significant dependence of the transient queue-size distribution not only on the utilization factor, but also on the shape of the batch size distribution (thus, in practice, on the variation of packet sizes). Of course, the essential dependence on the threshold value *N* is also visible. The stationary queue-size distributions (for m=1,⋯,8) are presented in Table 1 for two batch size distributions $P_{1}$ and $P_{2},$ and for ρ=0.75 and 1. The values were obtained by applying the well-known Tauberian theorem to Formula (34).

The results presented in Table 1 show that the sensitivity of the stationary queue-size distribution on the type of the batch size distribution is essentially visible for the lower value of the traffic load and it “expires” in the case of ρ=1. Moreover, clearly, the higher the threshold value *N*, the less likely a shorter queue is to be obtained.

By analyzing probability distributions visualized in Figure 1, Figure 2, Figure 3, Figure 4, Figure 5, Figure 6, Figure 7, Figure 8, Figure 9, Figure 10, Figure 11 and Figure 12 we can draw the following conclusions:

- The higher the threshold value *N*, the more volatile is the behavior of the appropriate transient probability;
- The behavior of probability distributions close to t=0 is characterized by greater variability with greater values of *m* (which is directly related to a buffer loading period starting at time t=0, during which it is possible to obtain higher values of the queue length);
- As can be observed, the graphs “stabilize” faster for a lower traffic load value ρ=0.75.

## 7. Conclusions

In this article, the explicit formula for the LT of the conditional queue-size distribution in the $M^{X}/G/1/K$-type finite-buffer queueing model with threshold-type control policy is derived. Analytical results are obtained by using an approach based on the construction of an embedded Markov chain, renewal theory and linear algebra.

In the literature, most of the results (particularly for the queue-size distribution) concerning models with limitations in access to the service station are obtained for the steady state of the system (in the case t→∞). In this paper, appropriate formulae are found in a compact form for the transient state of the system. The formulae are written directly, using the key parameters of the system and some recursively defined functional sequence, without defining any additional random walk.

Numerical examples are attached that illustrate theoretical results. In particular, the impact of the traffic load, batch size distribution and the threshold level *N* on the non-stationary behavior of the queue-size distribution are tested on examples and visualized in figures. The considered queueing model has potential applications as a mathematical model of energy-saving mechanisms in wireless computer and telecommunication networks, or in production systems.

## Figures and Tables

**Figure 4:**
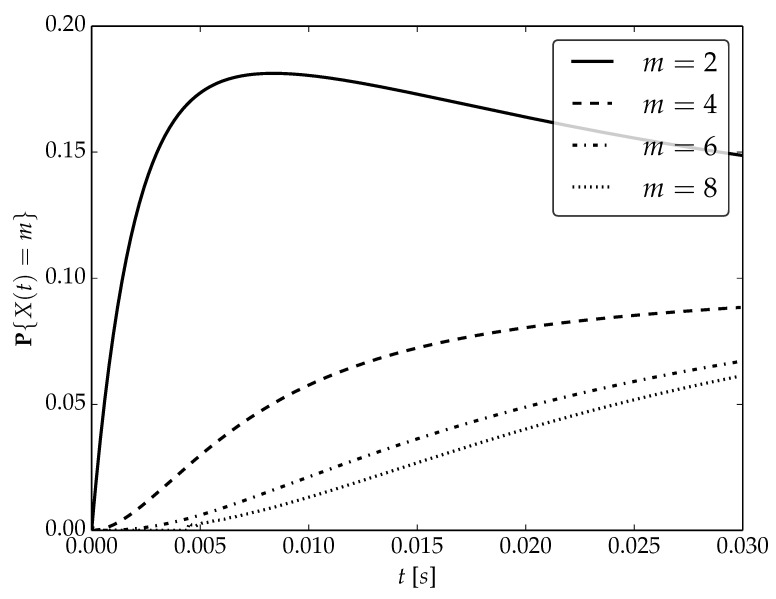
Transient distributions P{X(t)=m} for N=2, batch distribution $P_{2}$ and ρ=1.

**Figure 5:**
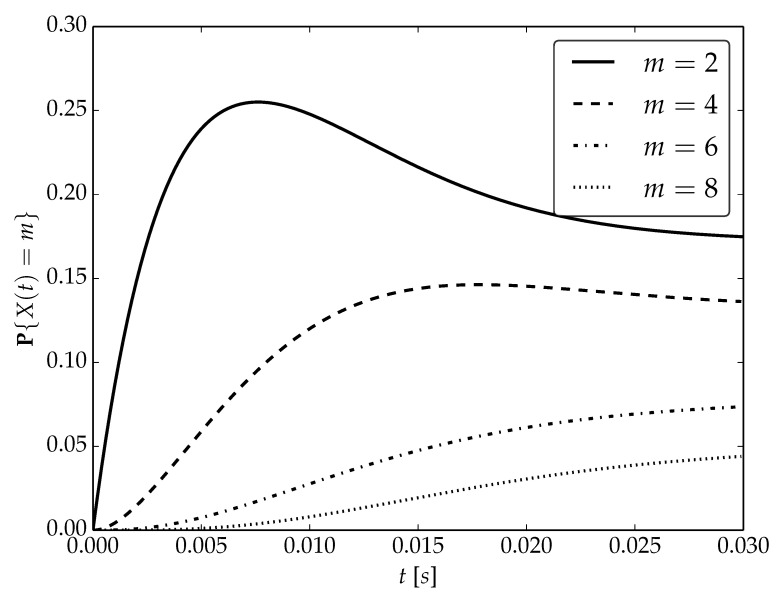
Transient distributions P{X(t)=m} for N=4, batch distribution $P_{1}$ and ρ=0.75.

**Figure 6:**
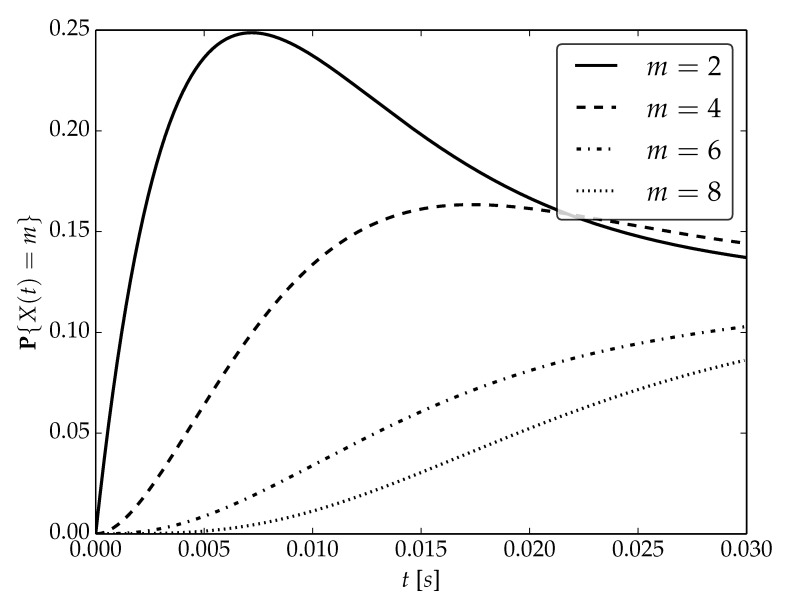
Transient distributions P{X(t)=m} for N=4, batch distribution $P_{1}$ and ρ=1.

**Figure 7:**
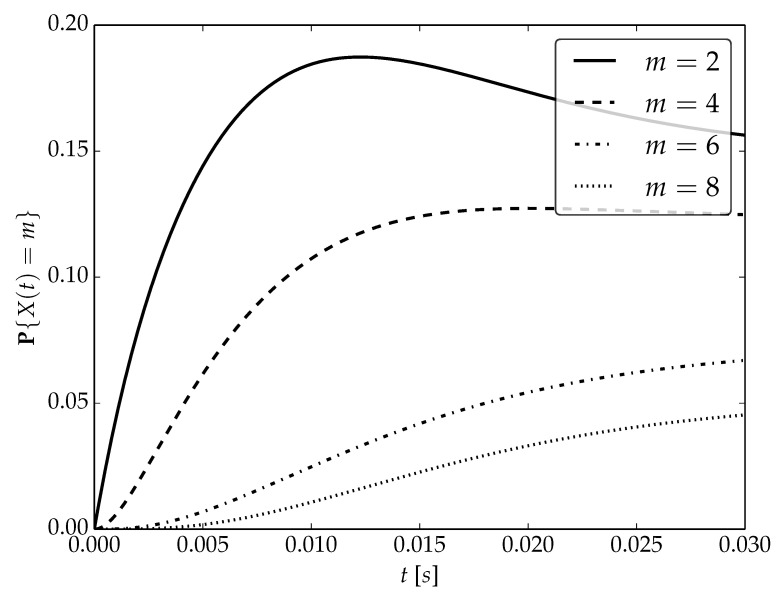
Transient distributions P{X(t)=m} for N=4, batch distribution $P_{2}$ and ρ=0.75.

**Figure 8:**
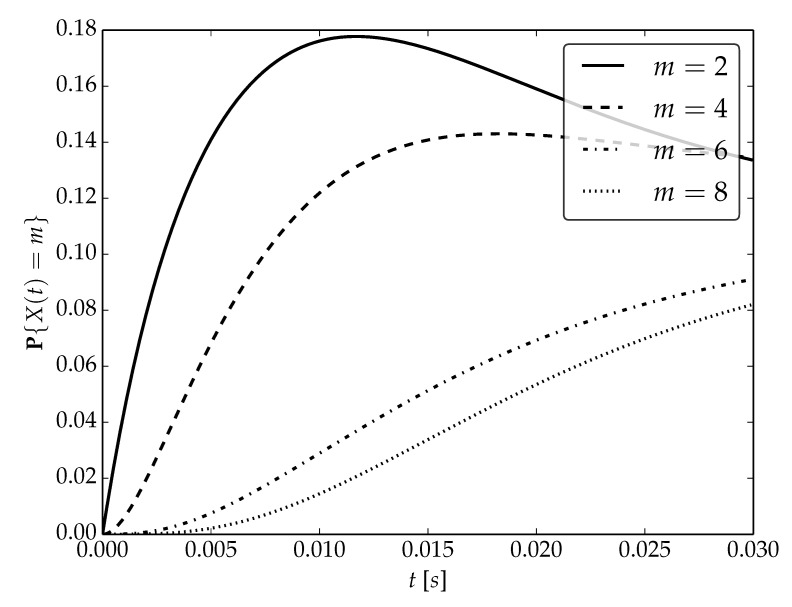
Transient distributions P{X(t)=m} for N=4, batch distribution $P_{2}$ and ρ=1.

**Figure 9:**
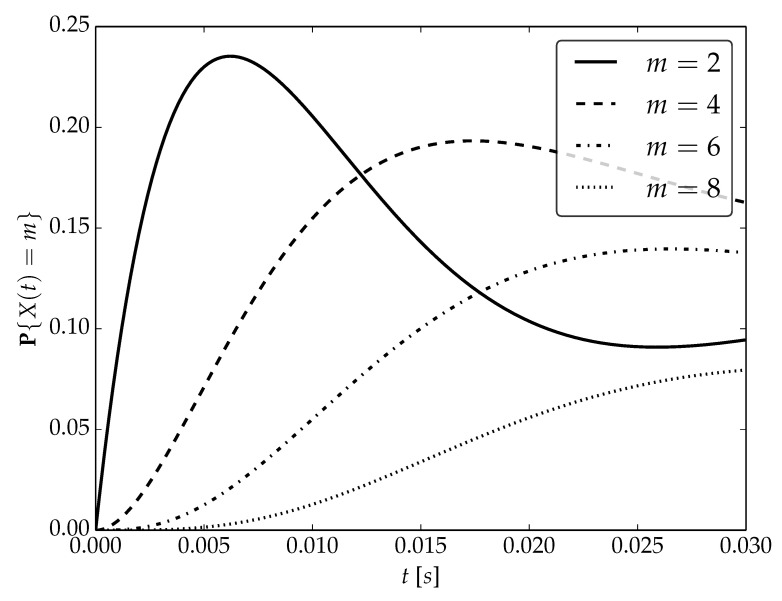
Transient distributions P{X(t)=m} for N=6, batch distribution $P_{1}$ and ρ=0.75.

**Figure 10:**
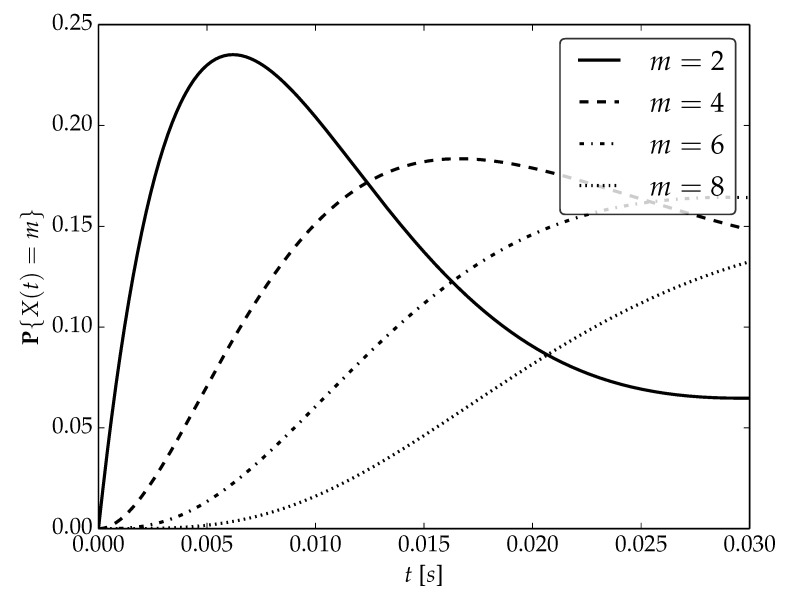
Transient distributions P{X(t)=m} for N=6, batch distribution $P_{1}$ and ρ=1.

**Figure 11:**
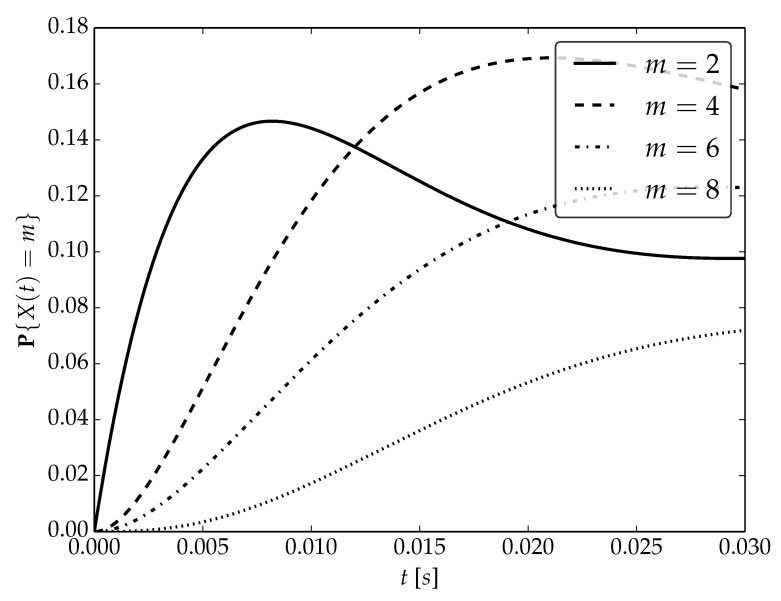
Transient distributions P{X(t)=m} for N=6, batch distribution $P_{2}$ and ρ=0.75.

**Figure 12:**
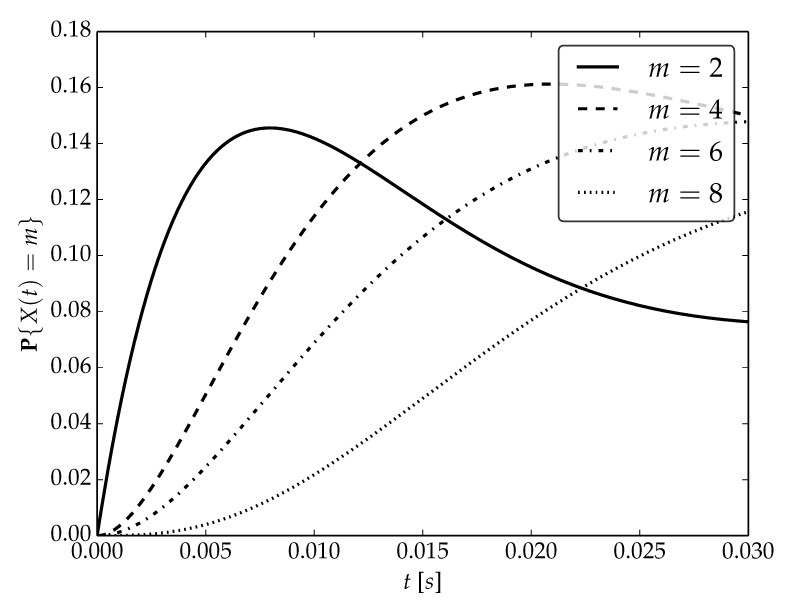
Transient distributions P{X(t)=m} for N=6, batch distribution $P_{2}$ and ρ=1.

**Table 1:** Stationary probability distributions P{X(∞)=m} for N=2,4,6, utilization factors ρ=0.75, ρ=1 and batch distributions $P_{1},P_{2}$.

	ρ=0.75		ρ=1	
m	$P_{1}$	$P_{2}$	$P_{1}$	$P_{2}$
N=2				
1	0.212075	0.199834	0.136161	0.137516
2	0.158953	0.145376	0.122422	0.123172
3	0.105852	0.080855	0.088267	0.0882393
4	0.092547	0.079795	0.098391	0.0981389
5	0.072634	0.068235	0.098877	0.0983271
6	0.059375	0.055587	0.096823	0.0960106
7	0.047794	0.047841	0.097677	0.0967295
8	0.038719	0.040349	0.097540	0.0965412
N=4				
1	0.112242	0.109196	0.077365	0.0774847
2	0.168366	0.148005	0.110890	0.111069
3	0.182401	0.193431	0.151724	0.151987
4	0.133278	0.123177	0.125696	0.125765
5	0.094683	0.081633	0.106656	0.106566
6	0.080649	0.074157	0.111316	0.111175
7	0.063987	0.063608	0.112137	0.111953
8	0.052152	0.052593	0.110932	0.110729
N=6				
1	0.080347	0.079517	0.058994	0.0590118
2	0.120510	0.107797	0.084558	0.0845924
3	0.130566	0.140905	0.115694	0.115754
4	0.150675	0.141098	0.128203	0.128264
5	0.160766	0.157617	0.152010	0.152073
6	0.118102	0.111993	0.138015	0.138019
7	0.086094	0.078620	0.123893	0.123844
8	0.072584	0.070182	0.127400	0.127354

## Data Availability

Not applicable.
